# The Role of NOTCH1, GATA3, and c-MYC in T Cell Non-Hodgkin Lymphomas

**DOI:** 10.3390/cancers14112799

**Published:** 2022-06-04

**Authors:** Mutaz Jamal Al-Khreisat, Faezahtul Arbaeyah Hussain, Ali Mahmoud Abdelfattah, Alhomidi Almotiri, Ola Mohammed Al-Sanabra, Muhammad Farid Johan

**Affiliations:** 1 Department of Haematology, School of Medical Sciences, Universiti Sains Malaysia, Kubang Kerian 16150, Kelantan, Malaysia; mutaz.alkhreisat@student.usm.my; 2 Department of Pathology, School of Medical Sciences, Universiti Sains Malaysia, Kubang Kerian 16150, Kelantan, Malaysia; faezahtul@usm.my; 3 Department of Medical Laboratory Sciences, Faculty of Applied Medical Sciences, The Hashemite University, Zarqa 13133, Jordan; alisharif@hu.edu.jo; 4 Department of Clinical Laboratory Sciences, College of Applied Medical Sciences—Dawadmi, Shaqra University, Dawadmi 17464, Saudi Arabia; hsalmutiri@su.edu.sa; 5 Department of Medical Laboratory Sciences, Faculty of Science, Al-Balqa Applied University, Al-Salt 19117, Jordan; ola.sanabra@bau.edu.jo

**Keywords:** GATA3, c-MYC, NOTCH1, NHL, PTCL, lymphoma

## Abstract

**Simple Summary:**

Peripheral T cell lymphomas (PTCLs) comprise 10–15% of all non-Hodgkin lymphomas, and are more aggressive with worse prognosis. Due to their rarity and significant molecular abnormalities, the pathogenesis of PTCLs is not well understood. The expressions of NOTCH1, GATA3, and c-MYC have been linked to a poorer prognosis in PTCL, and are implicated in downstream processes. The aims of this review are to elucidate the role of NOTCH1, GATA3, and c-MYC in patients with PTCLs to provide additional information about the disease’s pathogenesis and to investigate the master regulator between these genes that will provide a basis for new therapeutic strategies and improve PTCL patients’ survival rates. NOTCH1 greatly influences the progression, pathogenicity, and development of PTCLs. Because NOTCH signalling does not rely on enzymatic signal amplification, and all Notch ligands and receptors include extracellular domains that are essential for receptor binding and activation, making them accessible to circulating therapies and targeting for different therapeutic strategies.

**Abstract:**

Lymphomas are heterogeneous malignant tumours of white blood cells characterised by the aberrant proliferation of mature lymphoid cells or their precursors. Lymphomas are classified into main types depending on the histopathologic evidence of biopsy taken from an enlarged lymph node, progress stages, treatment strategies, and outcomes: Hodgkin and non-Hodgkin lymphoma (NHL). Moreover, lymphomas can be further divided into subtypes depending on the cell origin, and immunophenotypic and genetic aberrations. Many factors play vital roles in the progression, pathogenicity, incidence, and mortality rate of lymphomas. Among NHLs, peripheral T cell lymphomas (PTCLs) are rare lymphoid malignancies, that have various cellular morphology and genetic mutations. The clinical presentations are usually observed at the advanced stage of the disease. Many recent studies have reported that the expressions of NOTCH1, GATA3, and c-MYC are associated with poorer prognosis in PTCL and are involved in downstream activities. However, questions have been raised about the pathological relationship between these factors in PTCLs. Therefore, in this review, we investigate the role and relationship of the NOTCH1 pathway, transcriptional factor GATA3 and proto-oncogene c-MYC in normal T cell development and malignant PTCL subtypes.

## 1. Introduction

Lymphoma is a blood cancer that affects mature lymphocytes causing them to aberrantly proliferate and accumulate in the lymph nodes and throughout the lymphatic system [1]. Lymphomas are classified into two main types: Hodgkin (10% of cases), and non-Hodgkin lymphomas (NHL) (90%). NHL involves a heterogeneous group of over 60 lymphoproliferative malignancies with diverse patterns of behaviours and responses to treatments. NHL subtypes are defined based on the cell of origin either B lymphocytes (B cells, representing around 86% of NHL) or T cells and natural killer (NK) cells (T and NK cells, representing around 14%) [1,2,3]. Hodgkin lymphomas are primarily diagnosed based on the presence of Reed–Sternberg cells [4], whereas NHL is much less predictable than Hodgkin lymphoma, and its prognosis depends on the histologic type, clinical prognostic factors, and treatment received [5]. NHL was the 12th most frequent cancer in males and females worldwide with an estimated 544,352 new cases and 259,793 new deaths in 2020 [6].

Peripheral T cell lymphoma (PTCL) is a heterogeneous group of mature T and NK neoplasms that consists around 29 different subtypes according to the WHO Classification [1].PTCLs comprise about 10–15% of all NHLs and have aggressive behaviours and poor prognosis, more than most B cell NHLs [7]. Blood tests (including a full blood count and serum biochemical analysis), radiographic tests, and tissue biopsy are all part of the clinical evaluations. The pathologists face a difficult challenge in making a tissue diagnosis of PTCLs as the distinction between PTCLs are far-ranging in terms of cellular composition, morphological features and immunophenotypes. In addition, certain characteristics of various diseases overlap significantly, and not all diseases are linked to specific mutations. Furthermore, because of the rarity of and insufficient experience in diagnosing the disease, a number of reactive T cell lymphoproliferation can be misinterpreted as cancer. As a result, before rendering a final evaluation, pathology data must be incorporated considering the clinical [8]. The pathogenesis of PTCL mainly involves the dysregulation of TCR signalling pathways, interaction with the non-neoplastic tumour microenvironment and neoplastic transformation induced by viruses and chronic inflammation. In addition, PTCL oncogenesis includes gene mutations and cytogenetic abnormalities, such as chromosomal translocations, insertions, and deletions, which result in fusion proteins, constitutive activation, hyperactivation, and gene loss. The processes by which neoplastic lymphocytes mediate such a diverse variety of biological behaviours are based on a decrease in tumour cell immunogenicity and regulation of environmental signals and non-neoplastic cells. For tumour cell immunogenicity reduction, the expression of several costimulatory and regulatory proteins, including human leukocyte antigen class I and class II complex components, members of the B7 system (CD28), intercellular adhesion molecules (CD58 and LFA-1), and surface or intercellular regulators of apoptosis, can help PTCLs evade antitumor immune responses. T cell lymphoma can also evade immunological recognition by suppressing the immune response to proapoptotic stimuli. This effect is mediated by specific oncogenic events, such as the deregulation of extrinsic apoptotic pathways (Fas/FasL- and TRAL/TRAIL-receptor pathways), overexpression of antiapoptotic regulatory proteins (FLIP and AIP) and upregulation of the antiapoptotic and immune regulatory protein CD47 [9].

Immune escape strategies mainly depend on shaping the tumour microenvironment to promote neoplastic cell proliferation. Therefore, soluble mediators, such as cytokines and chemokines, are produced in T cell lymphomas. The cytokine milieu is a crucial grantor in lymphomagenesis. For example, PTCL-GATA3-enriched lymphomas induce the expression of T helper 2 cell (Th2) cytokines (interleukin [IL]-4, IL-5, and IL-13), PTCL-TBX1-enriched cases induce the overexpression of Th1-related molecules (IFNγ, CXCL12, CCL2, CCL3, CCL6, and CCL11), and T_FH_-enriched cases induce the expression of related molecules (CXCL13, IL-10, IL-2, IL-6, IL-17, and IL-8 angiogenesis) [10]. These soluble mediators also have an impact on the non-neoplastic microenvironment’s cellular constitution. PTCLs encompass a variety of non-neoplastic cells that play an important role in lymphomagenesis, including B and T lymphocyte subsets, tumour-associated macrophages (TAMs), eosinophils and endothelial cells. To reduce the antitumor immune responses, PTCLs selectively recruit non-neoplastic lymphocyte cells including regulatory T cells (Tregs) that play a significant role in these consequences. Adaptively induced Tregs have long been thought to play a role in oncogenesis by obstructing innate and acquired immune responses [11].

In PTCLs, all of the Treg subtypes have been addressed; for example, PTCL, NOS and anaplastic T cell lymphoma (ALCL) all have suppressor Tregs, but tumour-killing Tregs are linked with ENKTL subsets [12]. By targeting antitumor effector cells, PTCL cells can also govern cytotoxic immune responses. The aberrant expression of the immune-regulatory surface protein PD-L1 (B7-H1, CD274) blocks TCR-related signals, inhibiting effector T cell activation [13]. PD-L1 expression has been found to be higher in a variety of T-cell lymphomas, including AITL, ATLL, ALCL (anaplastic lymphoma kinase [ALK]-positive and -negative), ENKTL, MF, Sézary syndrome and PTCL-not otherwise specified (PTCL-NOS) [14,15,16]. TAM content is linked to poor prognosis, suggesting that this regulator has a direct role in tumour growth and progression [17]. Additionally, TAMs have an alternate tumorigenic (M2) phenotype that enhances the release of immune-modulatory cytokines (IL-10 and TGFα), the synthesis of proangiogenic factors and the expression of PD-L1 on the surface. Gene expression profile and whole-genome sequencing have revealed additional information that is valuable not only in recognising the various subtypes but also in giving insight into the pathogenies of PTCL [18]. Furthermore, mutational analyses are currently being used in clinical trials of new drugs to assess the various indicators of responses to enhance therapy decisions, such as using CD30-directed antibody drugs, CD25, CCR4 tag, PI3 kinase inhibitors, JAK/STAT inhibitors, and ALK inhibitors. In addition, extensive epigenetic dysregulation affects processes, such as the mutation in epigenetic regulators of KDM6A, MLL2, *TET2*, and *DNMT3* that govern genes in signalling pathways, such as *ZAP70*, *CHD8*, *APC*, and TRAF3 [19]. Gene expression studies of PTCL-NOS have identified two prognostic groups, each with a different presumed cell of origin. *GATA3* and its target genes (*CCR4*, *IL18RA*, *CXCR7*, *IK*) are expressed at high levels, and they are assumed to come from Th2-like cells and have poor prognosis. Additionally, GATA3 is considered as a master regulator of Th2 cell differentiation. Conversely, the expression of transcription factors TBX21 and EOMES, as well as their target genes (*CXCR3*, *IL2RB*, *CCL3*, *IFN*), are linked to a favourable outcome, and TBX21 is considered as a master regulator of Th1 cell differentiation. A small subset of TBX21-expressing PTCL with poor prognosis expresses cytotoxic markers and particular cytokine transcripts, such as CXCR3 and CXCL12, and has been linked to CD8+ cytotoxic cells [20,21]. Scientists explored the possible association between the presence of c-MYC, GATA3, and a high level of Ki-67 expression in PTCL patients to confirm whether the GATA3-positive subgroup of tumours is enriched in MYC and proliferative gene signatures compared with other groups. The results showed significant positive associations between c-MYC expression and the presence of both Ki-67 and GATA3, but no significant association between Ki-67 and GATA3. Furthermore, in the PTCL-NOS subgroup, a significant positive correlation was observed between c-MYC expression and the presence of both Ki-67 and GATA3. Enforced GATA3 expression in normal T cell development transforms double positive (DP) thymocytes into a pre-malignant state that is highlighted by elevated c-MYC expression, with subsequent stimulation of NOTCH1 signalling, which contributes to the establishment of malignant transformation [22]. The mRNA and protein expression of GATA3 had an inverse relationship with TBX21. In addition, GATA3 and TBX21 showed that GATA3 has poor overall survival compared with TBX21. The IFNα/β/γ regulated gene signatures, CD8+ T cell profile and NF-kB pathway signatures were significantly higher in the TBX21 subgroup than in the GATA3 subgroup, which had marginally higher mTOR- and MYC-related gene signatures and significantly higher PI3 Kinase-induced gene signatures [23].

In addition, extensive epigenetic dysregulation affects all these processes [19]. Recent studies have reported the role of NOTCH1, and the relationship of c-MYC and GATA3 expression with poor prognosis in PTCL [22,24]. c-MYC deficiency results in a drastic reduction in T cell proliferative capacity and cell growth [25]. There is also an emerging concept using GATA3 as a marker for the cell of origin in the diagnosis of PTCL-NOS as it predicts a worse prognosis [19]. In addition, NOTCH1 controls essential processes that are required for T cell development cell, growth, proliferation, differentiation, and apoptosis [22]. Therefore, understanding the key regulators of normal T cell development would facilitates the understanding of the lymphomagenesis of PTCLs. This review concentrates on the roles of NOTCH1, GATA3, and c-MYC in PTCLs. It is hoped that this review will be able to connect the relationship of these elements that affect the behaviour of PTCL, provide additional information in its pathogenesis and explore the master regulator between these genes to provide a basis for future therapeutic strategies and enhance the survival rates of PTCL patients.

## 2. Development and Regulation of T Cells

T cells are essential in controlling the immune response in the fight against infections and tumours. For example, tumour and virally infected cells are killed by the action of cytotoxic CD8 T Cells and their subtypes; CD4 T cell modulate the action of innate and adaptive cells (B and T cells) by secreting different types of cytokines that are necessary for the proliferation and differentiation of CD4 T cells into subsets of helper T (Th) cells: Th1, Th2, Th17, and Treg cells. As a result of antigenic and mitogenic stimulations, T cells go through a series of events that include cell activation, expansion, and differentiation [26].

**Figure 1:**
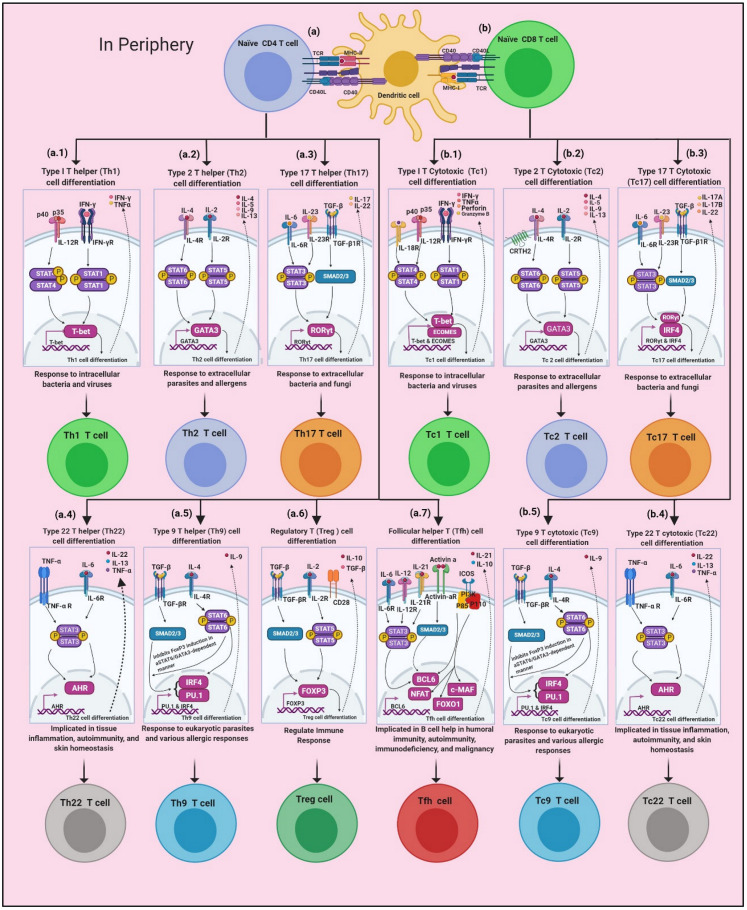
Molecular events involved in regulating T cell subtypes development: (**a**) T cell receptor (TCR) CD4 binds with major histocompatibility complex class II (MHCII); (**a.1**) Th1 cells being induced in response to the activation of specific intracellular INF-ϒ and IL-12 receptor pathways, that are mediated by STAT1 and STAT4, respectively; (**a.2**) Th2 cells are induced in response to mainly IL-2 and IL-4 receptor ligation and activation of STAT5 and STAT6 pathways, respectively; (**a.3**) Th17 cells are induced in response to activation of intracellular pathways of IL-6 or IL-21, and TGF-β receptors, and their activation mediated through STAT3 pathway for IL-6 or IL-21, and SMAD2/3 pathway for TGF-β; (**a.4**) Th22 cells are induced in response to IL-6 and TNF-α receptor ligation and activation of STAT3 pathways that lead to induction of Th22 master transcription factor AhR (the aryl hydrocarbon receptor); (**a.5**) Regulatory T cells (Treg) are induced in response to the activation of intracellular pathways of IL-2 and TGF-β receptors, that are mediated by STAT5 pathway for IL-2 and SMAD2/3 pathway for TGF-β; (**a.6**) Th9 cells are induced in response to activation of intracellular pathways of IL-4 and TGF-β receptors, that are mediated by STAT6 pathway for IL-4 and SMAD2/3 pathway for TGF-β; (**a.7**) T follicular helper (Tfh) cells are induced in response to activation of intracellular pathways of IL-6, IL-12, IL-21, and Activin A receptors, that are mediated by STAT3 pathway for Interleukins receptors, and SMAD2/3 pathway for Activin A receptor; (**b**) T cell receptor (TCR) CD8 bind with major histocompatibility complex class I (MHCI); (**b.1**) Tc1 cells induced in response to activation of intracellular pathways of INF-ϒ and IL-12 receptors, that are mediated by STAT1 and STAT4, respectively. As a result of STATs pathway activation, they lead eventually to induction of master transcription factor ECOMES and T-bet that encoded by the *Tbx21* gene, production of INF-ϒ, reinforces the Tc1 polarization, creating a positive feedback loop, and suppresses the alternative differentiation programs; (**b.2**) Tc2 cells induced in response to mainly IL-2 and IL-4 receptor ligations, and activation of STAT5 and STAT6 pathways, respectively. Appropriate STATs signalling leads to induction of master transcription factor GATA3, Tc2 polarization, antagonizes Tc1 polarization, and cytokines produced includes IL-4, IL-5, and IL-13, as well as effector molecules, such as granzymes and perforin; (**b.3**) Tc17 cells are induced in response to activation of intracellular pathways of IL-6 or IL-21, and TGF-β receptors, that is mediated by STAT3 pathway for IL-6 or IL-21 and SMAD2/3 pathway for TGF-β; (**b.4**) Tc22 cells are induced in response to activation of intracellular pathway IL-6 and TNF-α receptors, that are mediated by STAT3 pathway; (**b.5**) Tc9 cells are induced in response to activation of intracellular pathways of IL-4 and TGF-β receptors, that were mediated by STAT6 pathway for IL-4 and SMAD2/3 pathway for TGF-β.

There are numerous transcriptional and growth factors that regulate T cell differentiation and activation, and each subset of T cells has their own appropriate and master transcriptional factors (Figure 1).

**Figure 2:**
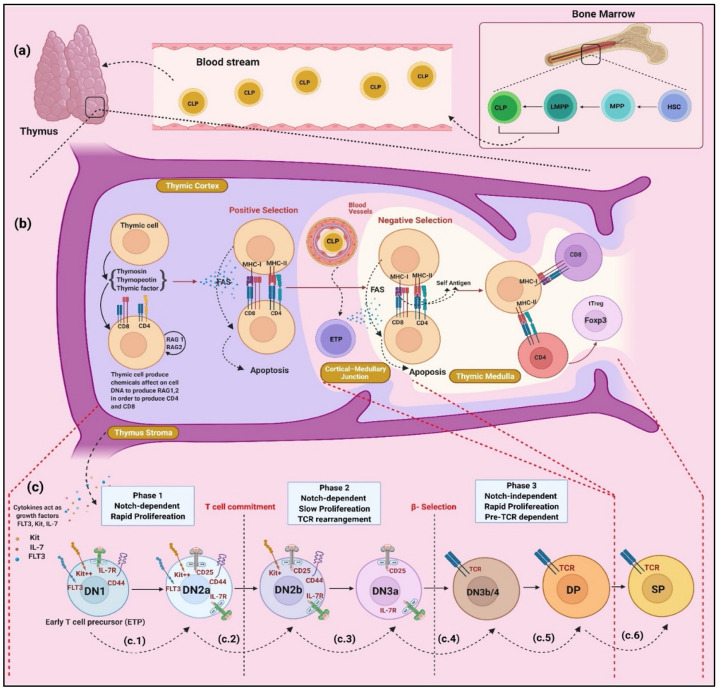
Niche of T cell selection and development. (**a**) In bone marrow (BM) hematopoietic stem cells differentiate to multipotent progenitor (MPP) that subsequently derived to subset called early lymphoid progenitors (ELP) or lymphoid primed multipotent progenitors (LMPP). LMPP differentiate into further downstream developmental stages, common lymphoid progenitor (CLP) that migrates from the bone marrow compartments into thymus through blood stream via the action of chemokines, cell adhesion molecules, and CCL25/CCR9, CCL19-CCL21/CCR7 receptors; (**b**) Cortical DP cells that express αβTCR interacting with MHC molecules presenting on edoncortical thymic epithelial cells (cTECs), receive critical survival signals that are required to further process of differentiation. In other words, T cells election and acquisition of MHC restrictions referred to as positive selection. Double positive (DP) cells begin expressing the chemokine receptor CCR7 and migrate to the thymic medulla. In the medulla, TCRs-DP T cells interact with antigen-presenting cells, such as mTECs and dendritic cells, and differentiate into CD4 or CD8 single-positive (SP). The interactions of high-affinity T cells TCRs to self-peptide MHC, normally result in either deleted of autoreactive cells by apoptosis that referred to negative selection or destined to become regulatory T cells through undergoing agonist selections; (**c**) Phases of early T cell development. ETPs in thymus undergo three distinct phases based on the status of T lineage commitment and NOTCH dependency; (**c.1**) Thymus stroma secrets cytokines that act as transcriptional factors include IL-7, FLT3, and cKit. These cytokines initiate the expression of some genes, such as *GATA3, Hes1*, and *Tcf*, through activation of NOTCH signalling pathway; (**c.2**) these genes beside *RUNX1* promote expansion of pre-commitment precursor as well as turn on transcriptional repressor Bcl11b in the late DN2a; (**c.3**) T cell passes from cell commitment and characterized with NOTCH-dependent, slow proliferation and TCR rearrangement. *BCL11B* turns on leads to c-Kit down-regulation, IL-7R signalling desensitization by E proteins, and DN2b cells survival become strictly NOTCH-dependent. E protein-dependent genes are recombination activating gene 1 (*Rag1*), *Rag2*, as well as *Ptcra* (encoding pre-TCRɑ), *Cd3e* (encoding CD3), and TCRβ (or TCRϒ and TCRδ) gene rearrangement; (**c.4**) Achieved V(D)J rearrangement for the *TCRβ* gene, express pre-TCR and the cells proceed to the DN3b stage after completion of β-selection, followed by transition into DN3/4 and cells gaining ability to receive signals through pre-TCR, transition from Notch-dependent to Notch-independent and leads to rapidly turning off Notch target genes and IL-7R expression; (**c.5**) Cells transition into DP (CD4^+^ CD8^+^); (**c.6**) TCRα gene in DP rearrangement differentiates into SP cells, expressing ɑβTCR, by the action of chemokine signalling, such as CXCL12/CXCR4, and up-regulation of new transcription factors; IKZF3 (Aiolos) and RORϒt.

The development of T cells occurs in the bone marrow (BM) and thymus (Figure 2a). Committed lymphoid progenitors (Lin^−^ Sca1^low^ c-Kit ^low^ IL7Rα^+^ FLT3^+^) [27,28] migrate from the BM to the thymus, where naïve T cells become functional and self-tolerant through positive selection in the thymic cortex and negative selection in the thymic medulla (Figure 2b). T cell development in the thymus undergoes three distinct phases based on NOTCH signalling pathways: NOTCH-dependent pre-committed (phase 1), NOTCH-dependent T-lineage committed (phase 2) and NOTCH-independent pre-TCR dependent (phase 3) phases (Figure 2c) [29]. In phase 1, because of the dense NOTCH ligands in the thymic cortical environment, the NOTCH pathway is activated once ETPs (c-Kit^high^ Lin^−^) enter the thymus. As a result, the NOTCH signalling pathway, in addition to transcription factors (Zeb1, Gata3, E2a, Tcf1, Bcl11b, etc.) and cytokines, play a critical role in controlling the fate of cell specification by regulating cell proliferation, differentiation, apoptosis, and survival [30,31]. Thymus stroma cytokines initiate the expression of some genes, such as *GATA3*, *Hes1*, and *Tcf* by activating the NOTCH signalling pathway. In addition to *RUNX1*, these genes promote the expansion of pre-commitment precursor and turn on the transcriptional repressor Bcl11b in the late DN2a [32]. Phase 2 of T cell development begins after the T cell has passed through cell commitment and is marked by slow proliferation and NOTCH-dependent and TCR rearrangement. As a result of the expression of Bcl11b, cells that have the potential to differentiate to non-T cell fates will be excluded [33]; DN2b cells downregulate c-Kit and become desensitised to IL-7 receptor (Il-7R) signalling by the mechanism mediated by E protein, and their survival is strictly NOTCH-dependent [34,35]. The NOTCH-dependent and E protein-dependent genes, such as recombination activating gene 1 (*Rag1*), *Rag2*, *Ptcra* (encoding pre-TCR), and *Cd3e* (encoding CD3), are expressed at their highest levels during the development of DN2b stage into DN3a stage [36,37,38]. After cells gain the ability to receive signals through the pre-TCR, the cells undergo a transition from NOTCH-dependent to NOTCH-independent in the early stages of phase 3 T cell development. However, cells still require NOTCH signals until they pass through β-selection [39]. Afterwards, NOTCH target genes become downregulated, whilst IL-7R is highly expressed [39] and become DN3b and then DN4 cells. Furthermore, by partially supporting chemokine signalling through CXCL12/CXCR4, DN4 cells enter a phase of extremely rapid proliferation, which is critical for complete phenotypic differentiation at the next stage [40,41,42]. In the final process of T cell differentiation, the cells not only express CD4 and CD8 co-receptors but also upregulate the expression of new transcription factors, including IKZF3 (Aiolos) and RORϒt, to allow for the silencing of the double negative (DN)-specific factors, such as ERG and HES1 [42,43].

## 3. NOTCH1 Signalling Pathways in T Cell Development and PTCLs

The NOTCH s pathway is highly conserved in mammals and plays an important role in regulating many developmental processes and in the maintenance of tissue homeostasis [44]. In normal T cell development, NOTCH controls the cell fate by regulating cell proliferation, differentiation, apoptosis, and survival [45]. The early stage of T cell differentiation is promoted by NOTCH signalling through the DN (CD4^−^CD8^−^) stage [46]. Through correlation with pre-TCR signalling, NOTCH mediates the transition into DP (CD4^+^ CD8^+^) stage [47]. In addition, NOTCH takes part in pre-TCR expression, TCRβ gene rearrangement, αβ versus γδ T cell decision, and the generation and migration of γδ T cells during T cell development [48]. There are four NOTCH receptors (NOTCH 1, 2, 3, and 4), consisting of heterodimeric transmembrane structure that are synthesised as a single protein which degrade proteolytically in the Golgi apparatus by furin-like protease at site S1 during transport to the cell surface [49]. NOTCH receptors in the extracellular domain contain 29–36 epidermal growth factor-(EGF-)like repeats (36 in NOTCH1 and NOTCH2, 34 in NOTCH3, and 29 in NOTCH4) that interact with NOTCH ligands (Delta-like 1 [DL1], DL3, DL4, Jagged1, and Jagged2). NOTCH-ligand interaction leads to the proteolytic cleavage of the transmembrane-intracellular domains of the receptors through ADAM metalloprotease, and the γ-secretase complex to generate an intracytoplasmic molecule of NOTCH (ICN). ICN then translocates into the nucleus, interacts with RBPJ DNA binding protein, and induces the targeted genes [50,51]. Moreover, the regulation of NOTCH signalling is mediated by NOTCH rapid degradation via proteasomal degradation of FBXW7, which is an E3 ubiquitin ligase that recognises PEST domain of ICN1, and finally leads to the termination of NOTCH1 signalling [52,53]. Furthermore, NOTCH regulates the expression of transmembrane receptors that operate upstream in the JAK/STAT and PI3K pathways, which are present in T lymphocytes, such as IL-7R, insulin-like growth factor 1 receptor, and pre-T cell antigen receptor alpha (PTCRA). These receptors play vital roles in the development of the early T cell, and their expressions are down-regulated through the course of differentiation [54,55].

In the hematopoietic system, NOTCH1 is strictly required for the commitment of primitive hematopoietic progenitors to the T cell lineage [56]. NOTCH1 signalling is identified in the pathogenesis of T-acute lymphoblastic leukaemia/lymphoma (T-ALL) by the activation of *NOTCH1* mutations in over 60% of T-ALLs; examples include the heterodimerisation (HD) domain mutation of *NOTCH1* found in 20% of T-ALLs, which leads to ligand-independent receptor activation [57]. PEST domain mutations, on the other hand, are detected in 15% of T-ALLs and result in increased ICN1 stability, which aberrantly prolongs NOTCH1 activation. Mutations in the *FBXW7* gene account for 20% of T-ALL cases, and because *FBXW7* mutations are linked to *NOTCH1 PEST* mutations, they increase ICN1 protein stability. Furthermore, because FBXW7 mutations are implicated in the degradation of other key oncoproteins, such as MYC, JUN, MCL1, and Cyclin E, they may be linked to additional oncogenic activities [58,59,60,61,62,63,64,65].

NOTCH1 is involved in T-ALL with translocation at t(7;9)(q34;34.3) and suggest that the rearranged form of human NOTCH homolog functions contributes to the transformation or progression in some T cell neoplasms [66]. The human NOTCH1 gene’s C-terminal region of EGF repeat 34 is positioned adjacent to the TCR promoter/enhancer in the t(7;9)(q34;34.3) translocation, resulting in the up-regulated expression of NOTCH1, that termed with translocation-associated NOTCH (TAN1) homolog [67]. As the recurrent chromosomal rearrangement of t(7;9)(q34;34.3) in human T-ALL is so rare that it accounts for less than 1% of the factors that play a role in the disease pathogenesis, scientists initially thought that NOTCH has a minor role in T-ALL pathogenesis. However, many recent studies found that more than 50% of human T-ALLs showed *NOTCH1* mutations in the extracellular HD and/or the C-terminal PEST domain that induce its functions. In addition, activating mutations of *NOTCH1* and its genes that modulate intracellular NOTCH1 turnover have been found in T-ALL mice models [57,59,60].

Some experimental evidence indicates that NOTCH pathways play a role in T-NHL development, for example, NOTCH1 expression is elevated in mouse ϒ-radiation-induced thymic lymphomas, whereas NOTCH2 expression is markedly decreased, suggesting that NOTCH2 inactivation is involved in T-NHL [68]. Furthermore, transgenic mice expressing NOTCH3′s constitutively active intracellular domain revealed a significant increase in thymocytes at the late DN cell stage, leading to T cell leukaemia/lymphoma [69]. NOTCH4 is expressed in both CD34^+^ and CD34^−^ populations in human BM cells. Experimentally, after Notch4-intracellular domain (Notch4-IC)-transduced cord cells were transplanted into mic, immature T cell engraftment was significantly greater, whereas B cell development was blocked. These findings suggest that NOTCH4 activation leads to increased stem cell activity, diminished differentiation, and altered lymphoid development [70].

NOTCH ligands also play an important role in the development, survival, and pathogenicity of T-NHL. By using immunohistochemistry (IHC) in classical Hodgkin disease and ALCL patient samples, Jundt F and his colleagues found that NOTCH1 proteins are highly expressed. Interestingly, Jagged1, a Notch1 ligand, is not only expressed in tumour cells but also in bystander cells neighbouring tumour cells that lead to activated NOTCH1 through homotypic or heterotypic cell–cell interactions, resulting in activated NOTCH1 and a dramatic increase in the growth of tumour cells, preventing cell apoptosis and affecting the tumour microenvironment [71,72]. NOTCH1 is expressed in nodal and cutaneous ALCL primary tumour tissues via an interaction with its ligand Jagged1 on ALCL cells which promotes tumour cell proliferation while preventing apoptosis [71,72]. Delta-like 4 is a NOTCH ligand that contributes to the regulation of NOTCH activity in endothelial cells during physiological and tumour angiogenesis [73]. The expression of Delta-like 4 in the tumour microenvironment and increased NOTCH3 signalling in tumour cells are related to the escaped of human T-ALL cells from dormancy [73]. However, Delta-like 4 neutralization suppresses tumorigenesis through reducing endothelial cell-mediated activation of NOTCH3 signalling in T-ALL cells [73].

NOTCH activation in T-ALL and T-NHL has stimulated the interest in using NOTCH signalling suppression as a therapeutic target in both diseases. The best agents to achieve this objective appear to be gamma secretase inhibitors (GSIs), which mediate the proteolytic cleavage that is crucial for NOTCH activation. However, most of T-ALL cell lines with NOTCH1 mutations, do not react to GSI treatment [74]. The loss of PTEN, which is transcription factor found in the endothelium and is required for NOTCH-mediated cell cycle arrest, has been identified as a key event leading to GSI resistance to NOTCH inhibition [74]. HES1 causes NOTCH1 to downregulate PTEN, resulting in Akt pathway upregulation, as the Akt pathway promotes survival and growth in response to extracellular signals. PTEN-deficient/GSI-resistant T-ALL cells move from *NOTCH1* to *Akt* as their oncogene of choice and are extremely susceptible to Akt pathway inhibitors [75]. Cyclin D3 is a direct target of NOTCH1 in peripheral and leukemic T cells that promote cell cycle progression and proliferation [76]. The expression of cyclin D3 and its catalytic partners cyclin-dependent kinase (CDK) 4 and CDK6 rescues the T-ALL cell lines DND-41, HPB-ALL, and T-ALL1 from G1 arrest caused by GSI [76]. Interestingly, cyclin D3 and CDK4 are overexpressed in NOTCH-dependent T cell lymphomas, indicating that cell-cycle inhibitors and GSI should be used together to treat T cell lymphomas [76]. Studies on primary PTCL biopsies showed that PTCL expresses the NOTCH1 receptor in vivo and exhibits evidence of NOTCH pathway activation, as proven by the nuclear localisation of active NOTCH1 in some cases [77]. This finding, along with the fact that NOTCH pathway genes are rarely mutated in PTCL, suggests that NOTCH signalling in PTCL might be mediated by mechanism other than DNA structural lesions, such as epigenetic deregulations or ligand-mediated activation from the microenvironment.

Furthermore, the inhibition of NOTCH by pharmacological blockers or small-interfering RNA causes apoptosis at advanced stages in primary cutaneous CD30 T cell lymphoproliferative diseases (primary cutaneous anaplastic large cell lymphoma and lymphomatoid papulosis), Sézary syndrome, and mycosis fungoides [72,78,79,80].

## 4. Roles of GATA3 in Normal T Cell Development and PTCLs

GATA family members play an important role in the proliferation and differentiation of hematopoietic and non-hematopoietic cells [81]. There are six members of the mammalian GATA family (GATA1 to GATA6), all of which include two zinc-finger motifs that are assumed to have arisen through gene duplication [82]. GATA family members have two distinct expression patterns: haematopoietic factors (from GATA1 to GATA3) and endodermal factors (from GATA4 to GATA6). GATAs include one or two C2-C2-type zinc-finger motifs, which interact with the GATA DNA sequence that is found in the regulatory regions of many genes included in targeted cells; for example, GATA3 protein binds to regulatory regions of genes that are necessary for Th2 differentiation and development [83,84], NK cell differentiation and function [85,86], Treg function [87], and differentiation and maintenance of type-2 innate lymphoid cells (ILC2) [88].

**Figure 3:**
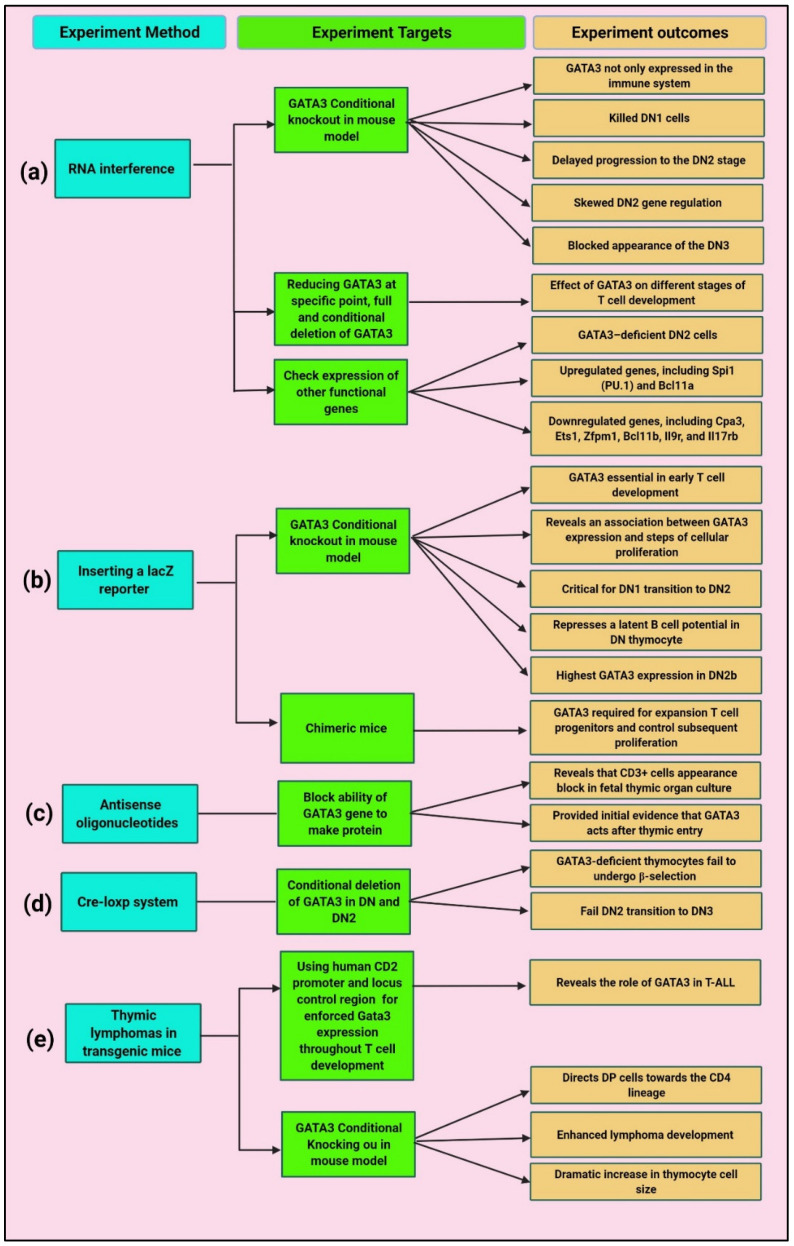
Experimental evidence of the determinate role of GATA3 in T cell development through using different experimental methods: (**a**) RNA interference used for reducing GATA3 during T cell development, inducing *GATA3* conditional knocking out in mouse model, and check gene expression of other functional genes; (**b**) Inserting LacZ reporter for inducing *GATA3* conditional knocking out in mouse model and chimeric mice generation; (**c**) Antisense oligonucleotide used for block ability of *GATA3* gene to make proteins; (**d**) Cre-loxp system used for *GATA3* conditional deletion of *GATA3* in DN and DN2; and (**e**) Thymic lymphomas in transgenic mice used for enforce GATA3 expression during T cell development, and *GATA3* conditional knocking out in mouse model.

GATA3 is expressed not only in the immune system but also in the embryonic and adult tissues, such as the adrenal glands, kidneys, central nervous system, inner ear, hair follicles, skin, and breast tissue in mice, with conditional knockout of GATA3 (Figure 3a) [89]. In immune cells, GATA3 functions as a master regulator of Th2 differentiation. It has also been found to be crucial for early T cell commitment, β-selection, and CD4^+^ T cell development and differentiation [90]. Gene targeting experiments revealed that GATA3 is essential in early T cell development. The experiments the *GATA3* gene was targeted through inserting a lacZ reporter by homologous recombination in embryonic stem (ES) cells (Figure 3b). Antisense oligonucleotides to Gata3 (ASO-GATA3) revealed that CD3^+^ cells appearance block in foetal thymic organ culture and thus provided initial evidence that GATA3 acts after thymic entry (Figure 3c) [91].

GATA3 is crucial in determining T cell precursor fates to eliminate B cell potential in DN stages 1 and 2 [92]. Determining the distributional binding site of GATA3 within variable stages of CD4^+^ CD8^+^ provided evidence that *GATA3* regulates different target genes at different stages of development [93,94]. Conditional deletion of *GATA3* using the Cre-loxp system in DN and DN2 cells revealed that Gata3-deficient thymocytes fail to undergo β-selection and DN2 transition to DN3, respectively (Figure 3d) [84,95]. *GATA3*-deficient cells fail to generate T cells in chimeric mice which emphasize that *GATA3* is required for the expansion of T cell progenitors and control of subsequent proliferation [84].

The overexpression of *GATA3* in T cells during development in thymic lymphoma directs DP cells towards CD4 lineage, enhances lymphoma development, and increases thymocytes size in CD2-Gata3 transgenic mice (Figure 3e). The developed monoclonal thymic lymphomas are CD3^+^, CD4^+^, and CD8^+/low^, suggesting a DP origin [95]. The enforced *GATA3* expression in DP cells of thymic transgenic mice T cells results in a premalignant state, and the cells showed increased size and C-MYC protein expression [22]. These cells also expressed higher levels of Hes1, a transcription factor that is a target of NOTCH1 signalling, revealing that NOTCH signalling may play a role in malignant transformation in *GATA3* overexpressing cells, potentially through c-MYC, which considered a direct target of NOTCH1 in T lymphoblastic leukaemia/lymphoma [22]. However, the relationship of GATA3 to other genes involved in T-ALL development in humans and mice, including the basic-helix-loop-helix (bHLH) proteins c-MYC, Tal1, and E2A; LIM-only domain proteins (Lmo1 and Lmo2); and the heterodimeric transmembrane receptor NOTCH1, is still unknown [96].

**Figure 4:**
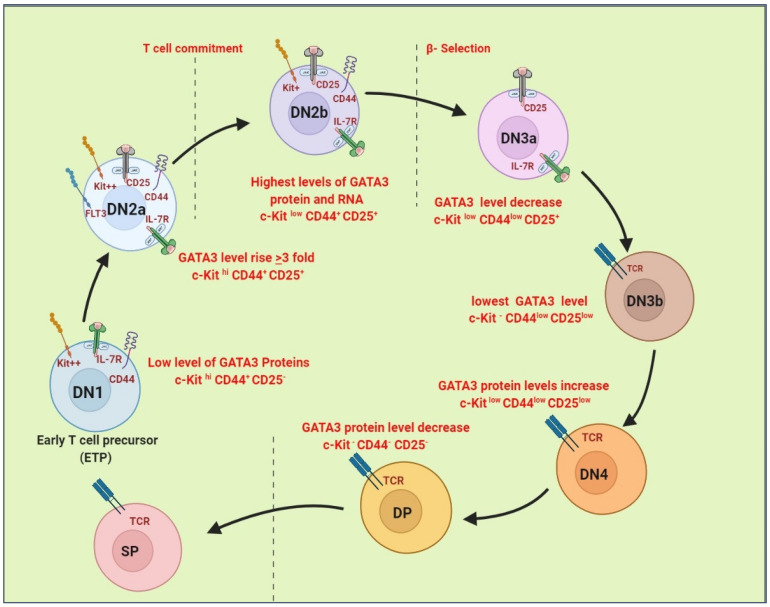
Expression of GATA3 during T cell development. Four-color intracellular detection of GATA3 in early DN thymocyte subsets from B6 adult mice they showed GATA 3 protein levels during T cell development besides defined subset expression of c-Kit, CD44, and CD25.

R. W. Hendriks and Scripture-Adams, D.D. expressed the effect of *GATA3* on different stages of T cell development through *GATA3* gene targeted by inserting a LacZ reporter through homologous recombination in embryonic stem (ES) cells and reducing *GATA3* at specific point by using RNA interference and conditional and full deletion (Figure 4). The results revealed that the highest *GATA3* expression in DN2b cell that pass from commitment stage and have lower expression of c-kit [95,96,97].

The GATA3 protein is initially low in the early T cell precursor (Kit^high^ CD44^+^ CD25) population of adult C57BL/6 mice. GATA3 levels marginally change as cells begin to express CD25 (‘DN1 25^low^’) but increase three-fold once CD25 is turned on to become DN2a (Kit^high^ CD44^+^ CD25^+^). When DN2b (Kit ^lower^) newly committed cells are separated from uncommitted cells by Kit expression levels [98], DN2b (Kit ^lower^) exhibits the highest levels of GATA3 protein and RNA. Before β-selection, the expression of DN3 cells (Kit^low^ CD44^low^ CD25^+^) is low, with the lowest expression in a separate fraction of DN3 cells with no detectable Kit expression (DN3 Kit^−^). Beginning with β-selection in T cells, GATA3 protein levels rise again in cells entering β-selection (DN3 25^low^) and through the DN4 stage (Kit^low^ CD44^low^ CD25^low^), but subsequently fall when the cells reach the DP (CD4^+^ CD8^+^) stage [99]. In the DN2 and DN3 stages, RNA levels, similar to protein levels, rise considerably.

## 5. c-MYC Roles in Normal T Cell Development and PTCLs

The *c-MYC* proto-oncogene, which is located on chromosome 8q24, is a basic helix-loop-helix leucine zipper (b/HLH/LZ) that regulates a diverse range of cellular functions, such as cell cycle, cell growth, survival, cellular metabolism and biosynthesis, adhesion, and mitochondrial function [100]. c-MYC is considered an essential global transcription factor regulating 10%–15% of all human genes, therefore c-MYC is tightly regulated at the transcriptional and translational levels [101,102]. Normally, c-MYC mRNA and c-MYC protein have very short half-lives in normal cells [103,104,105]. c-MYC needs appropriate positive regulatory signals because their proteins levels are low and insufficient to promote cellular proliferation [106]. In leukemogenesis, *c-MYC* has been recognised as a crucial direct downstream target gene of *NOTCH1*. NOTCH1 rescues T-ALL cells treated with GSI, although the overexpression of c-MYC is sufficient to rescue most human T-ALL cell lines from GSI-induced growth arrest [107].

The inhibition of NOTCH1 induces cell cycle arrest, reduces *c-MYC* mRNA levels, and prevents NOTCH1-mediated direct activation of c-MYC, which is crucial for leukemic cell survival [108]. The responsive region (TTCCCAA) of the c-MYC promoter localised between −195 and −11 bp binds NOTCH1 and its downstream effector CSL, result in upregulation of c-MYC at the mRNA and protein levels in malignant and non-malignant intracellular Notch1(ICN1)-overexpressing cells [109,110]. The deletion of c-MYC at the CD4^+^CD8^+^ stage of T cell development prevents tumour formation induced by NOTCH1 [111]. To investigate the role of c-MYC in leukemogenesis, a previous study altered NOTCH1 at the transmembrane domain after insertion mutagenesis in c-MYC transgenic mice, and revealed the high expression of truncated NOTCH1 RNAs and proteins [112].

## 6. NOTCH1 as a Bridge between GATA3 and c-MYC in T Cell Development

*NOTCH1* promotes IL-4 expression in CD4^+^ T cells which upregulate *GATA3* through the signal transducer and activator of transcription 6 (STAT6) cascade of the JAK/STAT pathway [113]. The absence of NOTCH1 signals leads to diminished GATA3 expression [113]. NOTCH1 acts in parallel with GATA3 to synergistically activate IL-4 expression, as shown in GATA3-deficient foetal liver HPC, which demonstrated an impairment in T cell specification [113,114]. As a result, NOTCH1 signalling is insufficient without GATA3 in the specification of T cell fate, and GATA3 and the intracellular region of NOTCH1 are essential in both T cell fate and development, and neither can use it alone [114].

*c-MYC* expression is induced directly through *NOTCH1*, and the inhibition of *NOTCH1* leads to cell cycle arrest, apoptosis, and reduced *c-MYC* levels [108]. In T-ALL and NOTCH1-induced transformation, MYC upregulation plays a key role in NOTCH1 oncogenic activity [115]. *c-MYC* is a direct downstream target of *NOTCH1* that contributes to the growth of T-ALL cells [108]. NOTCH signalling is required to maintain c-MYC expression in primary murine thymocytes at the DN3 stage of development [107].

In mouse tal1 tumour cell lines, leukaemia growth/survival remains dependent on the NOTCH1-c-MYC pathway; accordingly, the oncogenic functions of c-MYC and NOTCH1 have been established and a direct connection between NOTCH1 and c-MYC was revealed. Therefore, this finding supports the previous studies that found c-MYC to be a direct transcriptional target gene of the NOTCH1 pathway in T-ALL and c-MYC levels to mediate T cell transformation [116].

## 7. Conclusions

Many factors play vital roles in the progression, pathogenicity, incidence, and mortality rate of lymphoma. By exploring the factors that contribute to the development of lymphomas, we can direct the therapy to improve the overall survival rate and decrease the progression and development of lymphoma. NOTCH1 plays an important role in the regulation of GATA3 expression and c-MYC. Generally, after TCR ligation, IL-4-mediated activation of STAT6 through tyrosine phosphorylation plays an important role during Th2 cell differentiation and induction of GATA3. GATA3 is essential for early T cell commitment, β-selection, and the development and differentiation of CD4^+^ T cells. In addition to GATA3 a master transcription factor of Th2 differentiation, other transcription factors, including Zeb1, NFAT, NF-κB, and AP-1 family members play an important role in T cells differentiation. Moreover, in phase of T cell development, NOTCH signalling not only activates typical NOTCH target genes such as *Hes1*, but also begins the activation of T cell specification factor coding genes, such as *GATA3* and *Tcf7* (encoding TCF1 protein). The presence of a NOTCH/CSL binding site in the 3′ of the Il4 gene suggests that NOTCH signalling may directly control IL-4 production. Additionally, the NOTCH/CSL complex binds to the GATA3 gene’s distal promoter.

Experimentally, enforced GATA3 expression in DP thymic T cells resulted in a premalignant state, and the cells showed the increased size and C-MYC protein expression. In addition, these cells had higher levels of the transcription factor Hes1 suggesting that NOTCH signalling might play a role in malignant transformation in GATA3-overexpressing cells, presumably via c-MYC, which is a direct target of NOTCH1 in T lymphoblastic leukaemia/lymphoma.

NOTCH1 and c-MYC control two transcriptional pathways with shared target genes that work together to govern the proliferation of primary T cells. The inhibition of NOTCH1 induces cell cycle arrest, reduces c-MYC mRNA levels, and inhibits NOTCH1-mediated direct activation of c-MYC, which is required for leukemic survival. Furthermore, the NOTCH/CSL complex binds to the responsive element (TTCCCAA) of the c-MYC promoter that upregulates c-MYC in malignant and non-malignant ICN1-overexpressing cells at the mRNA and protein levels. Meanwhile, the deletion of c-MYC at the CD4+CD8+ stage of T cell development prevents tumour formation induced by NOTCH1. In leukemogenesis, studies have found that *c-MYC* is a crucial direct downstream target gene of *NOTCH1*. NOTCH1 rescues T-ALL cells treated with GS, although the overexpression of c-MYC rescues most human T-ALL cell lines from GSI-induced growth arrest. Interestingly, GATA3-positive tumours had higher levels of c-MYC and proliferation gene signatures than other PTCLs.

**Figure 5:**
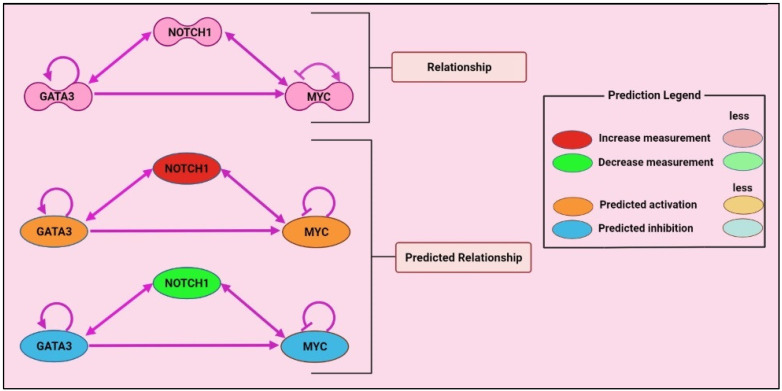
Relationship and predicted relationship of NOTCH1, GATA3 and c-MYC in T cell lymphoma development by using Ingenuity Pathway Analysis (IPA), QIAGEN.

Therefore, these data suggested that compared with GATA3 and c-MYC, NOTCH1 greatly influences the progression, pathogenicity, and development as well. In addition, NOTCH1 reveals a wide area for using different therapeutic strategies; for example, NOTCH signalling does not rely on enzymatic signal amplification like other signalling pathways, but rather on interactions between pathway components [117]. Thus, cellular regulatory mechanisms can precisely control signal intensity. Another critical characteristic is that the active form of NOTCH in the nucleus has a short half-life, which is critical for dynamic modulation of NOTCH signalling [118]. All Notch ligands and receptors include extracellular domains that are essential for receptor binding and activation, making them accessible to circulating therapies. To validate this prediction, through using Ingenuity Pathway Analysis, which is a Web-based software application from QIAGEN, and entering available data between these genes, it was predicted that down-regulation of NOTCH1 gene will lead to the downregulation of GATA3 and c-MYC, whereas the up-regulation of NOTCH1 leads to the up-regulation of these genes (Figure 5). Therefore, these findings support that NOTCH1 is more influence than other two genes in T cell non-Hodgkin’s lymphoma.

## 8. Future Directions and Therapeutic Targets

At present, the most common therapies for PTCLs are combinations of chemotherapy regimens, such as CHOP (cyclophosphamide, doxorubicin, vincristine, and prednisone), CHOEP (etoposide, vincristine, doxorubicin, cyclophosphamide, and prednisone), and brentuximab vedotin (Adcetris) in combination with cyclophosphamide, doxorubicin, and prednisone for CD30-expressing PTCLs. Usually, these regimens are provided with a high dose, especially after autologous stem cell transplant, because most PTCL patients relapse faster [7]. As a result of using these regimens, most PTCL patients face haematologic and non-haematologic toxicities that cause serious side effects, such as neutropenia, infectious complications, thrombocytopenia, septic shock and even death [119]. Although it was first described in classical Hodgkin’s lymphoma (cHL) and ALCL, CD30 expression is confined to medium/large active B and/or T-lymphocytes in normal or inflamed tissues [120,121]. The selective and dense CD30 expression on lymphomatous cells makes it an attractive target for drug-conjugated antibody-directed treatment, as their expression was reported previously in refractory cHL and later confirmed in animal models on ALCL [122,123]. In patients with CD30^+^ lymphomas, clinical studies with an unconjugated CD30-targeting monoclonal antibody (cAC10, SGN-30) showed safety but modest response rates. Therefore, laterally CD30-targeting monoclonal antibody bind with a monomethyl auristatin E to increase potency, selectivity, and led to delivery of cytotoxic agents to tumour cells (ADCs) [124]. Another therapeutic strategy used for targeting CD30^+^ lymphoma is by using chimeric antigen receptor T-cell immunotherapy (CAR-T). This can be accomplished by cloning CD30 extracellular fragment gene sequences from PTCL patients’ tumour tissues into a plasmid vector and expressing the CD30 antigen. Then, CD30 single-chain antibody fragment (scFv) will be created by using CD30^+^ monoclonal hybridoma cells generated from CD30 antigen-immunised mice [125]. Bispecific T-cell engager (BiTE) antibodies have the ability to redirect target cell lysis through T cells, trigger T lymphocyte killing in the presence of target cells (tumour cells), and allow T cells to lyse target cells [126,127]. The design of BiTE for CD30^+^ lymphoma cells will assist in directing the therapy and fighting lymphoma in a reliable way. Additionally, modifying the NOTCH pathway might be a useful tool for managing the transformed state of malignant lymphatic cells, potentially resulting in a novel treatment strategy. Therefore, the findings of this review will facilitate in exploring the controlling role of NOTCH1 amongst other genes in PTCL progression and development in order to be used in enhancing the overall survival rate and treatment plans, such as immunotherapy (CAR-T), ADCs and BiTE antibodies targeting against the *NOTCH1* gene. In addition, the most common method used for investigating the expression levels of these genes is IHC of PTCL tissue, which provides an easy way for monitoring the efficiency of the newly designed targeted therapy.
